# DNA Methylation Regulates Transcription Factor-Specific Neurodevelopmental but Not Sexually Dimorphic Gene Expression Dynamics in Zebra Finch Telencephalon

**DOI:** 10.3389/fcell.2021.583555

**Published:** 2021-03-19

**Authors:** Jolien Diddens, Louis Coussement, Carolina Frankl-Vilches, Gaurav Majumdar, Sandra Steyaert, Sita M. Ter Haar, Jeroen Galle, Ellen De Meester, Sarah De Keulenaer, Wim Van Criekinge, Charlotte A. Cornil, Jacques Balthazart, Annemie Van Der Linden, Tim De Meyer, Wim Vanden Berghe

**Affiliations:** ^1^Laboratory of Protein Chemistry, Proteomics and Epigenetic Signaling (PPES), Department of Biomedical Sciences, University of Antwerp, Antwerp, Belgium; ^2^Biobix: Laboratory of Bioinformatics and Computational Genomics, Department of Data Analysis and Mathematical Modeling, Ghent University, Ghent, Belgium; ^3^Department of Behavioral Neurobiology, Max Planck Institute for Ornithology, Seewiesen, Germany; ^4^Bio-Imaging Lab, Department of Biomedical Sciences, University of Antwerp, Antwerp, Belgium; ^5^Laboratory of Behavioral Neuroendocrinology, GIGA Neuroscience, University of Liège, Liège, Belgium; ^6^Laboratory of Pharmaceutical Biotechnology, Faculty of Pharmaceutical Sciences, Ghent University, Ghent, Belgium

**Keywords:** DNA methylation, zebra finch, brain development, epigenetics, song learning, dosage compensation, gene expression

## Abstract

Song learning in zebra finches (*Taeniopygia guttata*) is a prototypical example of a complex learned behavior, yet knowledge of the underlying molecular processes is limited. Therefore, we characterized transcriptomic (RNA-sequencing) and epigenomic (RRBS, reduced representation bisulfite sequencing; immunofluorescence) dynamics in matched zebra finch telencephalon samples of both sexes from 1 day post hatching (1 dph) to adulthood, spanning the critical period for song learning (20 and 65 dph). We identified extensive transcriptional neurodevelopmental changes during postnatal telencephalon development. DNA methylation was very low, yet increased over time, particularly in song control nuclei. Only a small fraction of the massive differential expression in the developing zebra finch telencephalon could be explained by differential CpG and CpH DNA methylation. However, a strong association between DNA methylation and age-dependent gene expression was found for various transcription factors (i.e., *OTX2*, *AR*, and *FOS*) involved in neurodevelopment. Incomplete dosage compensation, independent of DNA methylation, was found to be largely responsible for sexually dimorphic gene expression, with dosage compensation increasing throughout life. In conclusion, our results indicate that DNA methylation regulates neurodevelopmental gene expression dynamics through steering transcription factor activity, but does not explain sexually dimorphic gene expression patterns in zebra finch telencephalon.

## Introduction

Like humans, songbirds are one of the few animal groups that learn vocalizations (Jarvis, 2019). Vocal learning requires coordination of auditory input and vocal output using auditory feedback during a critical neurodevelopmental stage. Whereas female zebra finches (*Taeniopygia guttata*) lack the ability to sing, males learn their vocalizations from a tutor in early life, analogous to humans. They form an auditory memory template of the tutor song, to which they match their own vocalizations. This sexual dimorphism is reflected in the song control system, a series of interconnected telencephalic nuclei, which undergoes striking changes in morphology and connectivity during the song learning period. Given the many parallels with human (speech) learning, song birds are a commonly used model for neurobiological research (Drnevich et al., 2012; Brainard and Doupe, 2013).

At the basis of these developmental changes lies a highly complex and strictly regulated process, orchestrated by the coordinated expression of thousands of genes. Although the development of the song control system is highly sensitive to steroid hormones, hormones are not sufficient to explain the sex differences. Instead, intrinsic genetic mechanisms are thought to initiate sexual differentiation in the zebra finch song circuit, and sex-linked genes may play key roles (Arnold, 1997; Wade and Arnold, 2004; Chen et al., 2005; Tomaszycki et al., 2009). Additionally, the structure and function of the developing brain are also altered by sensory and motor experience, as well as parent–child relationships and stress (Kolb and Gibb, 2011). Which specific genes are involved in the genetic mechanisms controlling sexual differentiation, and their relationship with steroid hormones, still remains unclear. Nevertheless, genes located on the W but also the Z chromosome are of particular interest, given that males are the homogametic sex in birds (ZZ, vs. females, ZW) and that dosage compensation is much more limited than in mammals (Ellegren et al., 2007; Itoh et al., 2007, 2010).

Besides, the environment has an especially potent and long-lasting influence on the development of brain circuits during early life “critical periods.” These are phases of enhanced plasticity during which environmental input is required for a correct development of a particular brain circuit (Hensch, 2005). To date, our understanding of the dynamic transcriptional programs, the underlying molecular mechanisms (e.g., transcription factors involved), and the interaction between genes and environment that occur in concert with the song learning period remains largely unknown. In mammals, CpG dinucleotide methylation is known to facilitate diverse environment-induced biological processes, such as cellular differentiation (Kinde et al., 2015). In brain cells, dynamic changes in DNA methylation occur in response to neuronal activity at specific loci in the genome (Tognini et al., 2015), and recent studies also revealed the accumulation of non-CpG and hydroxymethylation during early postnatal neuronal development (Kinde et al., 2015; Stroud et al., 2017; Price et al., 2019). DNA (promoter) methylation also regulates gene expression in zebra finches (Steyaert et al., 2016), yet little is known about its function in song learning.

The goal of the present study was to gain understanding of the sexual differentiation and developmental programs in the zebra finch brain at the transcriptional level, as well as of their epigenetic regulation during the song learning period. Hence, we have analyzed gene expression (RNA-Seq) and DNA methylation profiles (reduced representation bisulfite sequencing, RRBS) in male and female telencephalon samples at days 1, 20, and 65 of postnatal development and in adults. One day post hatching (1 dph), where song nuclei are not yet visible, precedes the critical period as well as the onset of sexual differentiation of the song system (Wade and Arnold, 2004). At 20 dph, morphological (and sexual) differentiation of the neural song system is well underway, and birds are beginning to form a memory template of their tutor’s song (start of the sensory phase). At post-hatching day 65, the sensory memorization phase has ended (father’s song is memorized), and male birds are in the middle of the sensorimotor learning phase, during which they learn to copy their father’s song (Bottjer et al., 1985). In adults, the song control system is fully mature, and the male song has become crystallized.

Our results point toward transcription factors under epigenetic control regulating developmental gene expression. Additionally, sexually dimorphic gene expression was found to be regulated by an incomplete dosage compensation largely independent of DNA methylation. The generated data are publicly available (GEO ID: GSE147974), thereby creating a valuable resource for future research.

## Materials and Methods

### Animals and Tissues

For RNA-Seq and RRBS experiments, zebra finches (*T. guttata*) were bred and raised at the University of Liège in an aviary containing 20 nest boxes maintained in a 13:11 h light/dark cycle. Birds were provided *ad libitum* with food, water, grit and cuttlebone, and additional millet branches and egg food. Sprouted sunflower seeds were provided twice and nesting material was provided once per week. The experiment was performed according to Belgian law on animal experimentation and approved by the Ethical Committee for Animal Experimentation at the University of Liège (protocol 1396). Brains from male and female juvenile and adult zebra finches [age: 1, 20, 65 (±3 days), and adults (>120 dph)] were used (9 birds per sex/age combination, total *n* = 72), with birds from one nest randomly assigned to different age groups. Birds were rapidly decapitated, and the brains immediately removed and handled under ribonuclease-free conditions. The telencephalon was separated from the rest of the brain. The brain tissue was rapidly frozen on dry ice and stored at –80°C until further processing.

For immunofluorescence experiments, additional male zebra finches were bred in an aviary colony at Antwerp University (Ethical approval ECD 2016-70) under a 12:12 h light/dark cycle. They were provided with *ad libitum* food and water, egg food, cuttlebone, grit, and nesting material. Brains were collected at the ages of 20 and 120 dph (*n* = 4 per time point), as described before (Majumdar et al., 2014). Birds were deeply anesthetized using ketamine–xylazine solution (0.003 ml/g body weight) and transcardially perfused with ice-cold saline (pH 7.4) followed by 4% paraformaldehyde in 0.1 M phosphate buffer (pH 7.4) for fixation. Brains were quickly dissected out and postfixed overnight in the same fixative. Next, brains were cryoprotected by immersing them sequentially in 10, 20, and 30% sucrose solution (Merck) in 0.1 M phosphate buffer at 4°C until they sank in the solution. Brains were then embedded into 15% PVP solution (polyvinylpyrrolidone; PVP40T, Sigma) and stored at –80°C until further processing. The sex of the birds, initially identified based on gonadal morphology, was confirmed using PCR as described previously (Soderstrom et al., 2007).

### Experimental Design

To assess differential expression and methylation in the telencephalon during zebra finch postnatal development, RNA-Seq and RRBS analyses were performed. For each sex per time point (1, 20, and 65 dph and adult), three independent biological pools were sequenced, each pool consisting of RNA/DNA of three telencephalons in equal proportions, resulting in a total of 24 pools. Pooling was performed to increase power by reducing interindividual variation unrelated to the research hypotheses at hand. To avoid confusion, each pool will be labeled as a “sample” throughout this manuscript. As the same 24 samples were used for both RNA-Seq and RRBS analyses, expression data can thus be directly correlated with DNA methylation.

### DNA and RNA Isolation

Telencephalon tissue was lysed in QIAzol^TM^ lysis reagent (Qiagen 79306) using a TissueLyser instrument (Qiagen 85300). RNA was extracted using the RNeasy mini kit (Qiagen 74106), according to the manufacturer’s instructions. Subsequently, the combined interphase and organic (phenol) phase fractions of QIAzol^TM^ reagent-lysed material were used for genomic DNA isolation. In short, ethanol was added to precipitate the DNA. The DNA pellet was then washed with sodium citrate solution (0.1 M sodium citrate in 10% ethanol). Next, the pellet was incubated with 75% ethanol after which the pellet was dried and resuspended in DNase- and RNase-free water.

### RNA Sequencing

Quality control and library preparation for RNA sequencing was performed by the NXTGNT sequencing facility of Ghent University (Ghent, Belgium). RNA concentrations were quantified and quality controlled using the Quant-iT^TM^ Ribogreen^®^ RNA Assay (Invitrogen R11491) kit and Agilent RNA 6000 Nano (Agilent 5067-1511; on Agilent Bioanalyzer). Upon DNase treatment, library preparation (TruSeq stranded mRNA, Illumina RS-122-2101 and RS-122-2102) was performed on 1 μg of RNA. As recommended by Illumina, fragmentation was performed for 8 min at 94°C. RNA was pooled (as described in the experimental design), and subsequently, multiplex identifiers were added to each pool (“sample”). To increase uniformity of the protocol, library preparation was performed using the IP-Star Compact Automated System (Diagenode B03000002). Bioanalyzer QC after library preparation (Agilent, High Sensitivity DNA kit) demonstrated no relevant batch effects. Single-end 75 bp sequencing was performed three times for each of the pooled libraries on the Illumina NextSeq500 (over four lanes).

### (Oxidative) Reduced Representation Bisulfite Sequencing

RRBS (Meissner et al., 2005) was also performed by NXTGNT. DNA was purified using Zymo’s Genomic DNA Clean and Concentrator^TM^-10 to avoid inhibition of enzymatic digestion. The Quant-iT^TM^ PicoGreen^TM^ dsDNA Assay Kit (Invitrogen P11496) was used to assess the DNA concentration. After pooling in equal proportions, the resulting 24 pools (“samples”) were dried and redissolved in water (molecular grade) to obtain a concentration of 2 μg and subsequently digested using *MspI* (New England BioLabs R0106, 20,000 units/ml). After purification of the digestion product with a GeneJET PCR Purification Kit (Thermo Fisher Scientific K0701), the Qubit 2.0 Fluorometer (Q32866) was used to determine the concentration.

For the library preparation, the NEBNext^®^ Ultra^TM^ DNA Library Prep Kit (New England BioLabs, E7370S) was used. For each sample, half of the DNA was oxidized for oxidative RRBS (oxRRBS), while the other half was subjected to regular RRBS analysis. Subsequently, bisulfite and oxidative bisulfite conversions were performed using the TrueMethyl Seq kit (Cambridge Epigenetix, CEGX). A PCR with TrueMethyl DNA Polymerase was performed as a bisulfite conversion control and showed no aberrations. RRBS sequencing was performed on the Illumina NextSeq500. As initial paired-end experiments (2 × 76 nt) demonstrated low quality of reverse reads, only forward reads were used, and subsequent experiments relied on single-end sequencing (1 × 76 nt). Also, low-diversity issues led to overclustering and lower sequencing efficiency. Therefore, in collaboration with Illumina, different numbers of dark cycles were used to optimize the resulting useable library size (see Supplementary Table S1). Additionally, different concentrations of control PhiX were used as a function of other libraries (independent of this project) sequenced during the same run. Given that samples were randomized over different sequencing pools and the optimization particularly focused on increasing yield (forward read data quality was similar and acceptable for all experiments), no bias and, at most, a limited increase in technical variance is expected due to these variations in the protocol.

### Sequence Read Mapping and Summarization

The Zebra Finch reference genome as provided by Ensembl (assembly taeGut3.2.4, release 87) was used for mapping of both RNA-Seq and (ox)RRBS sequencing reads. FastQC (v0.10.1) was used to assess RNA-Seq data quality, indicating no relevant problems. Therefore, no additional trimming was performed. STAR (v2.5.2b) was used to align and map reads to the reference genome (Dobin et al., 2013). Samtools (v0.1.18) and htseq-count (v0.6.0) (based on GTF file, Ensembl, assembly 3.2.4, release 87) were used for downstream analysis and data summary, respectively.

For RRBS, quality control and filtering of low quality reads was performed using “Trim Galore!” (Babraham Bioinformatics). Quality control indicated no relevant problems, so Bismark (v0.9.0, Babraham Bioinformatics) was used in Bowtie2-mode (Krueger and Andrews, 2011) for mapping (Supplementary Table S2). Seed length, mismatches, and interval during multiseed alignment were set to the default values. Hydroxymethylation was determined by subtracting counts from total methylation (i.e., methylation and hydroxymethylation specific) RRBS with oxidized methylation (i.e., methylation specific) RRBS results. However, hydroxymethylation counts were too low for an accurate locus-wise analysis (signal not distinguishable from noise for individual loci, see Supplementary Figure S2). Furthermore, total methylation RRBS counts were used for methylation quantification since oxidized methylation RRBS results showed less consistent coverage. The data were subsequently imported using the Bioconductor BiSeq package (Hebestreit et al., 2013) for extraction and summarization of CpG and CpH methylation values, resulting in, respectively, 270,754, and 447,351 sufficiently covered dinucleotides. Ensembl (assembly taeGut3.2.4, release 95) and UniProt annotation (R-package UniProt.ws, v2.14.0) were used for annotation of transcription start sites (TSS), start and end positions of genes, and obtaining gene symbols for Ensembl gene IDs throughout all analyses.

### Differential Gene Expression Analysis

For normalization and differential gene expression analysis, the R package edgeR (Robinson et al., 2010) with its GLM functionality (v3.8.6, R version 3.6.1) was used, as this gave higher quality results (better normalization according to the MA plot, more uniform *P*-value distribution, data not shown) than DESeq2 or limma-voom. Normalization was performed using trimmed mean of *M* values (TMM). Genes with very low expression [<1 counts per million (cpm)] in 22 or more samples were filtered out. A stagewise approach as described by Van den Berge et al. (2017) was performed. Firstly, an omnibus test was used to check for all contrasts of interest simultaneously, yielding Benjamini–Hochberg-based false discovery rate (FDR) estimates. Subsequently, *post hoc* tests were performed [adjusted for multiple testing by calculating the familywise error rate (FWER) per *post hoc* analysis] for initially significant loci (FDR < 0.05). The age effect was assessed by comparing the average effect over 20 and 65 dph and adult birds to the expression at 1 dph. The sex effect was assessed at each age separately.

### Differential DNA Methylation Analysis

For the RRBS data, raw counts were used to calculate methylation percentages (β-values) and subsequently *M*-values, with offset α equal to 0.01. Though originally developed for Infinium HumanMethylation BeadArray data (Du et al., 2010), *M*-values can also be applied on methylation sequencing data (Sun et al., 2015). The main advantage of *M*-values is that they are more appropriate for use with the R/Bioconductor limma package (v3.22.7), which uses moderation to obtain realistic variance estimates thereby increasing the degrees of freedom (and thus power) (Ritchie et al., 2015).

(1) $$\beta =\frac{\#\,M\,e\,t\,h\,y\,l\,a\,t\,e\,d\,r\,e\,a\,d\,s}{\#\,T\,o\,t\,a\,l\,r\,e\,a\,d\,s}$$

(2) $$M=\log_{2}\left(\frac{\beta +\alpha }{1-\beta +\alpha }\right)\left(\alpha =0.01\right)$$

Given the intrinsic normalization accomplished by the method of calculation for β and *M*-values, no additional normalization was performed. The data were filtered to improve quality: (i) only loci that had a minimal average coverage of 5 × per sample and (ii) a minimum of six methylated reads over all samples combined were retained for further analysis. Limma was applied to the quality-filtered data to ensure proper mean-variance trend estimation, but when solely focusing on the subset of CpGs for differentially expressed genes, FDRs were recalculated accordingly for this subset (using Benjamini–Hochberg’s method). Differentially methylated CpGs were annotated as “genic” when located within gene start and end position in Ensembl annotation unless the CpG was also within the promoter region of that gene, then it was annotated as “promoter.” In all other cases, CpG dinucleotides were annotated as “intergenic.” Promoter sequences were defined as the genomic regions from 2,000 bp upstream to 500 bp downstream of the TSS (similar for transcription factor binding analysis, see section “Materials and Methods”).

### Visualization of Genomic Features in the UCSC Genome Browser

For visualization and integration of different data sources, results are presented as Zebra Finch (taeGut2) genomic feature tracks, compatible with the UCSC genome browser. RRBS results were compiled as bedGraph files: for significantly differentially methylated cytosines, the methylation percentage is displayed as a positive value (between 0 and 1), whereas for non-significant cytosines, negative values and a different color are used. To visualize RNA-Seq data, the bamCoverage function (version 3.4.3) from the deepTools suite was used to obtain coverages per 50 bp bin (default). RNA-Seq tracks, which are provided as BigWig files, were rescaled to take into account differences in sample size.

### Hierarchical Cluster Analysis

Dendrograms were created using the hclust function in R, using the complete linkage clustering algorithm on the covariance between samples. For RNA-Seq, the normalized log-transformed cpm after filtering was used, whereas for RRBS, we opted for *M*-values after filtering on coverage and minimal methylation counts as mentioned above. For computational reason, only the 10,000 most variable *M*-values were used for clustering of the RRBS data. As only 13,891 genes were retained after filtering of the RNA-Seq data, the whole dataset was used.

### Functional Enrichment Analysis

To identify biological processes significantly associated with differentially expressed/methylated genes during zebra finch postnatal development, we used two different platforms: Genomatix Pathway system and Metascape. Only Gene Ontology (GO) biological processes identified by both platforms were reported. The first platform that was used for enrichment analysis is the Genomatix Pathways System (GePS) Software (Intrexon Bioinformatics Germany), Release 2.10.0, Database ElDorado 04-2019, Genomatix Literature Mining 02-2019. This software uses information from public and proprietary databases as well as co-citations in the literature to enable a comprehensive analysis of enriched biological processes (GO terms). As this system is based on human data, zebra finch genes were converted to the corresponding human orthologs using BioMart (Ensembl Genes 91). Only pathways with adjusted *P* < 0.05 were considered. In addition, we used the web-based Metascape analysis resource (Zhou et al., 2019) (updated version 2019-08-14)^1^ (Zhou et al., 2019). For each given gene list, the Metascape pathway and process enrichment analysis were carried out using default settings, using only GO biological processes. For the age effect on gene expression, the 3,000 most significantly differentially expressed genes were used as input gene list.

A custom background gene list was used for both Genomatix and Metascape enrichment analyses. For RNA-Seq data, the background list consists of all genes that were considered for differential expression analysis (filtered for minimal expression). For RRBS, all genes that were sufficiently covered in the RRBS analysis were used as background. Figures were made based on the Genomatix GePS results.

### Transcription Factor Binding Site Enrichment Analysis

Transcription factor (TF) binding site (TFBS) enrichment analysis was performed using JASPAR (JASPAR 2018 server) (Mathelier et al., 2016), an open source, curated database of binding motifs for transcription factors. Probability weight matrices (PWM) and annotation were downloaded for analysis. As avian TFBS PWMs are practically unavailable, TF binding specificities are generally well conserved (Nitta et al., 2015), and we study zebra finches as a model for human learning and selected all human TF (from the non-redundant TF set for vertebrates). For RNA-Seq data, promoter sequences of significant differentially expressed genes were scanned with the PWMs using FIMO (v4.11.3) (Grant et al., 2011). For computational reasons, FIMO was run for each PWM separately. Subsequently, all matches (*P* < 1.0E-4, FIMO default) were retained, counted, and listed.

A chi-squared test was performed per TF binding motif to determine whether the respective motif was enriched or depleted between promoter regions for genes of interest compared with an outgroup. For expression-based TF analysis, genes that were not significantly differentially expressed for any of the investigated contrasts were selected as an outgroup. For the expression pattern analysis, all sufficiently covered genes not present in a given expression pattern cluster were used as an outgroup. Finally, for dosage compensated genes (either from birth or acquired, see further), non-dosage compensated genes were used as an outgroup. Benjamini–Hochberg correction for multiple testing was applied on the *P*-values obtained from the chi-squared tests. For the differentially methylated CpGs, the same procedure was performed but on the 100-bp flanking regions (50 bp upstream, 50 bp downstream) of each differentially methylated CpG in promoter regions. As outgroup, the flanking sequences of non-differentially methylated CpGs in promoter regions were used.

### Classification of Age-Dependent Gene Expression Patterns

Since broad trends in gene expression data may point to shared major biological mechanisms, log-transformed cpm values were used to assess changes in expression patterns between different ages. Therefore, the expression at the age of 1 dph was used as a reference, and the expression of a gene at a certain age was considered increased or decreased if more than 1.65 standard deviations, respectively, higher or lower compared to 1 dph (standard deviation was calculated for each variable separately). This way, assuming a normal distribution, about 5% of genes are expected to exceed this expression threshold (one-sided increase or decrease, depending on the trend evaluated). Considering three different levels—a lower (L), equal (E), or a higher/elevated (H) expression at three time points (20 and 65 dph and adult compared to 1 dph), 27 possible gene expression patterns are possible. As a symbolic notation, these contrasts are referred to by the relative state at the three time points, e.g., genes that exhibit an elevated expression from 20 dph onwards (i.e., higher expression at 20 dph, higher expression at 65 dph, and higher expression at adult) will be referred to as HHH. From all possible gene expression patterns, 12 patterns were selected: having a different expression at only one of the time points (LEE, ELE, EEL, HEE, EHE, and EEH), at 20 and 65 dph (LLE and HHE), from 20 dph onwards (LLL and HHH), or from 65 dph onwards (ELL and EHH).

### Integration of Gene Expression and DNA Methylation Results

Association between expression and methylation was evaluated by means of linear mixed models (lmerTest R package, which relies on the lme4 package) calculated for each differentially expressed gene with at least one remaining CpG after the RRBS filtering steps. Log-transformed cpm values were modeled as a function of the average *M*-value of the remaining CpGs for the given gene and sample, with gene and sample as random factors (random intercepts). This was done for CpGs for both promoter and genic region subsets separately. Therefore, only CpGs with a unique gene annotation were considered for this analysis (±80% of data remaining).

To further investigate the role of DNA methylation on gene expression, a chi-squared test was used to evaluate whether the distribution of differentially methylated CpGs was different in differentially expressed genes compared with non-differentially expressed genes. Additionally, for the differentially expressed genes, the percentage of genes containing at least one differentially methylated CpG was calculated. Regulation of gene expression by CpH methylation was evaluated analogously.

### Immunofluorescence

Male zebra finches were sampled at 20 and 120 dph (*n* = 4 per group). An antibody specific for methylcytosine (5mC) (clone 33D3, Active motif; cat no. 39649) was used to detect DNA methylation in zebra finch brain slices by means of immunofluorescence (IF). For IF, paraformaldehyde-perfused brains in PVP molds (see higher, “*Animals and Tissues”* section) were sectioned sagittally at 18 μm using a cryostat (Thermo Fisher Scientific^TM^ CryoStar NX50). Sections were collected on Thermo Scientific SuperFrost charged slides. Antigen retrieval was performed in PBS-T (PBS with 0.1% Triton X-100)-washed sections using 10 mM sodium citrate buffer for 3 min. Sections were blocked in 20% bovine serum albumin (BSA) in PBS for 1 h at room temperature (RT) and incubated with primary antibody (1:100) for 2 h at RT and overnight at 4°C. The next day, sections were incubated with secondary antibody (Texas Red, donkey anti-mouse IgG, Abcam; ab6818) for 2 h at RT, mounted in ProLong Gold mounting media with DAPI (Thermo Fisher Scientific) and visualized using an Olympus BX51 fluorescence microscope equipped with an Olympus DP71 digital camera. Olympus Cell-F Software was used for image acquisition. Images in both UV and Alexa Fluor 555 range were taken at 40× magnifications keeping the exposure constant for all sections. Pictures were taken for the song nuclei HVC (formerly “hyperstriatum ventral pars caudalis” and “high vocal center”), RA (robust nucleus of the arcopallium), Area X, and LMAN (lateral magnocellular nucleus of the anterior nidopallium), as well as an equally large area just outside each nucleus, as shown in Figure 7A.

(ImageJ) version 1.52 (Ferreira and Rasband, 2020; University of Chicago, 2020). The nuclear intensity of 5mC expression was quantified using 40× fluorescent images as described in ImageJ user guide for basic intensity quantification. The images, taken at fixed exposure, were converted to grayscale and thresholded using the triangle method. 5mC signal intensity was measured for 15–20 individual cell nuclei per brain area considered. For each brain region (HVC, RA, Area X, LMAN), the effect of age (20 vs. 120 dph) and inside/outside the nucleus (song nuclei compared with an equally large area just outside the nuclei, as shown in Figure 7A) was evaluated by means of linear mixed models (lmerTest R package), with brain slice (eight in total) as a random factor. A *P* < 0.05 was considered significant.

### Sexually Dimorphic Gene Expression

To assess differences between distributions of autosomal or sex chromosome-related genes, a chi-squared test was used. A pairwise comparison between four time points leads to six chi-squared tests, for which *P*-values were Bonferroni corrected for multiple testing. A similar approach was used to assess whether rates of overexpression in male birds compared with overexpression in female birds were different over time points for either autosomal or sex chromosome-related genes.

For GO and TFBS analysis, Z chromosome genes were divided into genes overexpressed in male birds and genes overexpressed in females. Since, for Z-linked genes, female overexpression is quite rare, genes that show overexpression in female birds for at least a single time point were categorized as overexpressed in female birds (independent of the behavior at other time points).

### Dosage Compensation Analysis

Cpm values were used to evaluate the dosage effect. Ratios of male (M) vs. female (F) cpm values (M:F ratios) were calculated per time point for autosomal and Z-linked genes separately. Genes located on the autosomal or Z chromosomes for which the exact location is not known (indicated with “_random” in the Ensemble annotation) were included in the respective groups [impact of removing them was negligible, data not shown; note that also the pseudoautosomal region (PAR) is located on such a “random” fragment]. Based on the M:F ratio distribution for autosomal genes (i.e., without dosage compensation), the 95% quantile was used as cutoff for dosage compensation, leading to three groups of Z-linked genes: (i) dosage compensated genes from birth, M:F < cutoff at 1 dph and at adult age; (ii) genes that acquire dosage compensation throughout the birds’ life, M:F > cutoff at 1 dph, but lower at adult age; and (iii) non-dosage compensated genes, M:F > cutoff at both 1 dph and adult age. For the graphical representation of these ratios, 1,000 bins were created within the range of ratios to calculate the percentage of genes in each bin. Subsequently, loess regression was performed to smoothen the produced plots.

To estimate differences in average expression between different dosage compensated genes and non-dosage compensated genes, the log-transformed cpm value for each gene was averaged over all samples. Each variable was then attributed to its respective class (dosage compensated from birth-acquired dosage compensation–no dosage compensation), and ANOVA (with *Tukey post hoc*) analysis was used for comparison. The same type of analysis was used to assess the influence of sex and age on methylation in (non)dosage compensated genes.

## Results

### Age- and Sex-Specific Changes in Telencephalon Gene Expression During Neurodevelopment

Gene expression at 1, 20, and 65 dph as well as in adults was measured for three samples per sex (each sample being a mix of three biological replicates) by RNA-Seq. On average, 57.4 (± 7.2) million reads (minimum of 42.8 million) were obtained per sample; mapping percentages (using STAR) were on average 81.05% (± 0.95%) supporting high-quality data (Supplementary Table S3). Upon data summary and filtering (see “Materials and Methods” section), 13,891 genes were analyzed for the 24 samples. As shown in Supplementary Figure S1, the expression data cluster by age, with the 1– and 20-dph time points being most different from the other time points, while the differences between 65 dph and adult birds are less apparent. Sex-based RNA-Seq clustering was more accurate for younger specimens.

The majority of genes (10,193; 73.4% of genes surveyed) exhibited a significant effect of age on expression (Supplementary Table S4). Important examples include the very significant (all adj. *P*–value < 3.0E-25) and relevant effects observed for the expression of parvalbumin (10.4-fold increase with increasing age), *GAD67* (*GAD1*) (3.3-fold increase), and *OTX2* (27.3-fold decrease), which are crucially involved in the timing of critical periods (Bernard and Prochiantz, 2016).

A smaller number of genes (1,157; 8.3% of genes surveyed), of which 48.1% were located on the Z-chromosome, exhibited a significant sex effect for at least a single time point (Supplementary Table S4). Interesting examples of differentially sex-specific expressed genes include *ESR2* (chr 5), *GADD45G* (chr Z), and *NTRK2* (chr Z). *ESR2* exhibited higher expression in females at 1 dph [fold change (FC) 3.6] and is the only sex hormone receptor exhibiting sexually dimorphic expression. *GADD45G*, involved in regulating learning-induced immediate early gene expression (Apulei et al., 2019), is differentially expressed at 1 and 20 dph, but not at the later time points. *NTRK2* (also known as trkB) encodes the receptor for neurotropic factors *BDNF* and neurotrophin 4 (*NTF4*) and is expressed at a higher level in males compared with females, yet—interestingly—not in adult birds.

**FIGURE 1:**
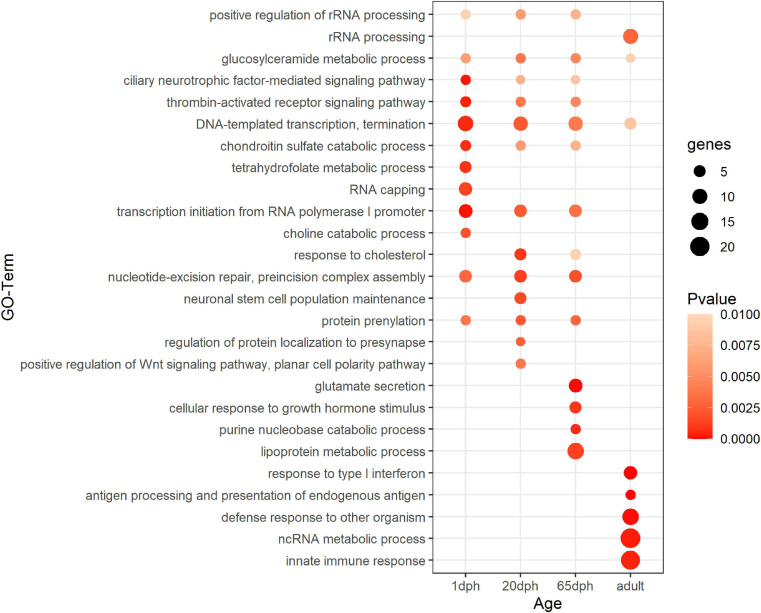
Functional enrichment analysis for sex-biased genes. Summary of statistically enriched GO biological processes for sex-biased genes at each assayed time point. This is based on Genomatix Pathways System (GePS) functional enrichment analysis. The dot size reflects the number of genes enriched in the Gene Ontology (GO) term and its color the corresponding statistical significance (*P*-value). Its absence indicates the annotation is not significantly enriched at the given time point. Complete enrichment analysis results are included in the (Supplementary Table S5).

Functional enrichment analysis of sex-biased genes showed significant enrichment for DNA-templated transcription termination and glucosylceramide metabolic processes for all time points (Figure 1 and Supplementary Table S5). Genes differentially expressed at 1 dph exhibited additional enrichment for RNA capping, as well as several metabolic processes (tetrahydrofolate, folic acid, choline, etc.). Genes differentially expressed at 20 dph were specifically enriched for the GO terms response to cholesterol, neuronal stem cell population maintenance, protein localization to presynapse, and positive regulation of Wnt signaling pathway, which were not enriched at any other time point. Genes exhibiting sexually differentiated expression at 65 dph showed enrichment for glutamate secretion. Finally, genes that were sex-biased in adults featured enrichment for response to type I interferon and innate immune responses (Figure 1 and Supplementary Table S5). Interestingly, we observed significant transcriptional changes of various epigenetic and epitranscriptomic writer–reader–eraser proteins in an age- or sex-dependent manner across neurodevelopment (Supplementary Table S6).

TFBS analysis of the bulk set of all genes differentially expressed with age revealed enrichment of various important neurodevelopmental TFBS including *EGR3* (Pfaffenseller et al., 2018), *REST* (Thiel et al., 2015), and *CTCF* (Hirayama et al., 2012; Watson et al., 2014), although the fold change of enrichment was limited (| log FC| < 1; Supplementary Table S7). *REST* and *CTCF* significantly change in expression over development themselves, which was not the case for *EGR3* (Supplementary Table S4). Similarly, we next screened for TFBS motifs near the TSS of sex-biased genes (Banerjee et al., 2019; Heberle and Bardet, 2019). TFBS enrichment analysis showed significant enrichment for many TFBS (Supplementary Table S7) including several ETS, POU, and FOX family transcription factors, although these TFs did not show major sex-specific changes in expression themselves.

In the next paragraph, we will further elaborate on age-dependent gene expression patterns, whereas sex-specific gene expression effects will be discussed in more detail in subsequent sections on dosage compensation.

### Age-Dependent Gene Expression Pattern Groups

**FIGURE 2:**
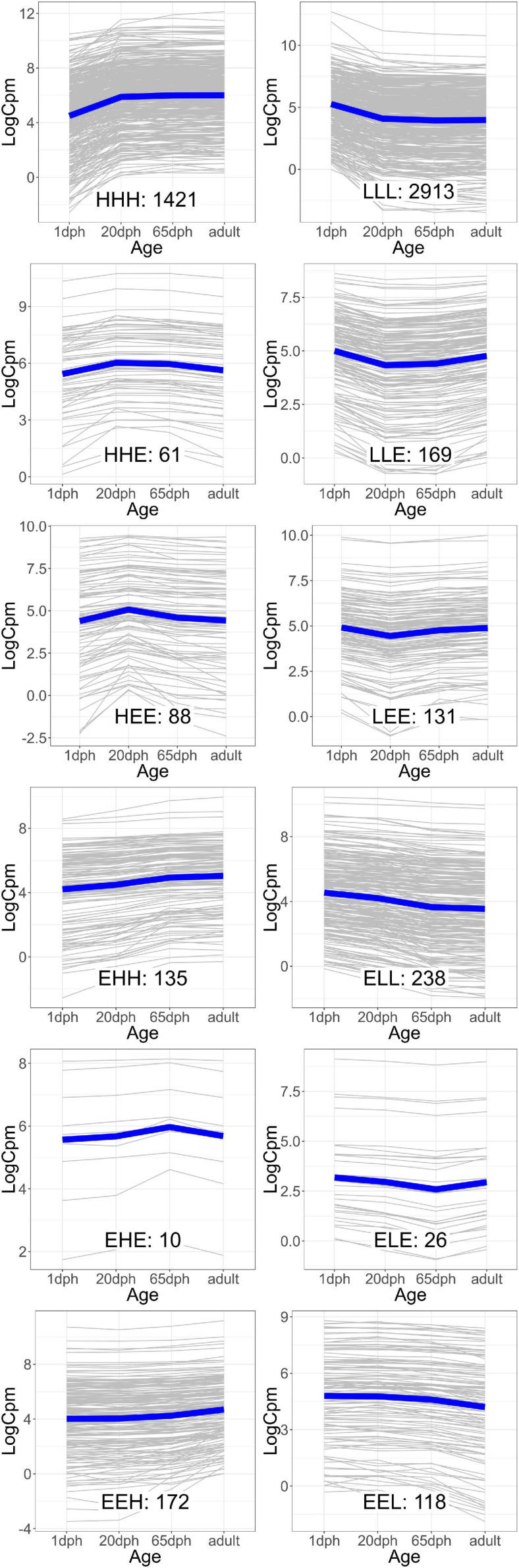
Groups of genes with similar gene expression patterns over the four time points. The expression of each individual gene [in counts per million reads mapped (cpm)] is plotted in gray, while the blue line indicates the average trend. The number of genes in each group is indicated. Group names reflect the relative expression at each time point [20 and 65 days post hatch (dph) and adult] compared with 1 dph, with H, higher, E, equal, and L, lower.

**FIGURE 3:**
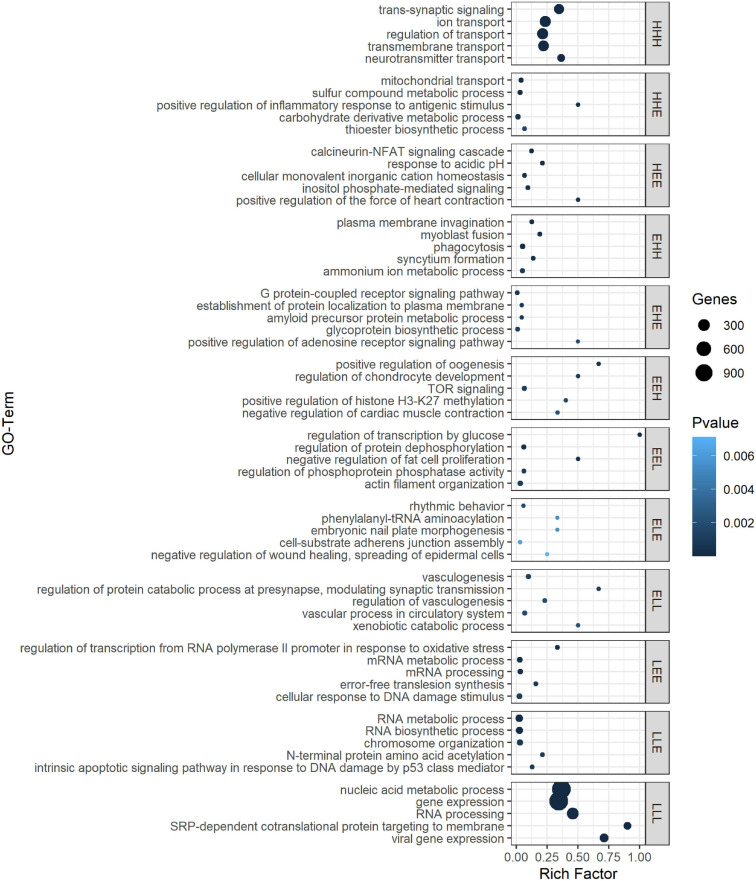
Top 5 ranked GO biological processes for the different gene expression pattern groups. GO terms are sorted by *P*-value in GePS functional enrichment analysis. For similar GO terms, only the top-ranked GO term was shown. The dot size reflects the number of genes enriched in the GO term and its color the corresponding statistical significance (*P*-value). Rich factor is the ratio of differentially expressed gene numbers annotated in this GO term to all gene numbers annotated in this GO term. Complete enrichment analysis results are included in Supplementary Table S9). H, higher, L, lower, and E, equal compared with 1 dph as reference.

As the age-dependent gene expression differences described in the previous section are likely the result of a combination of underlying mechanisms, genes were classified into groups with similar developmental patterns. In total, 12 gene groups, covering a total of 5,482 genes (39.5% of those with sufficient coverage), showing one of the specific developmental patterns of interest [combinations of equally (E), higher (H), and lower (L) transcription at each time point relative to 1 dph], were analyzed (Figure 2 and Supplementary Table S8). Functional enrichment analysis (Supplementary Table S9) and TFBS enrichment analysis (Supplementary Table S10) indicate that several of these groups reflect coordinated, large-scale cellular changes during brain development. A summary of the functional enrichment results can be found in Figure 3.

For example, we found a clear enrichment in genes involved in synaptic signaling, ion and neurotransmitter transport, and behavior for group HHH, which contains 1,421 genes that are lowly expressed at 1 dph, yet feature higher expression for the three later time points (hence, HHH, cf. “Materials and Methods” section) (Supplementary Table S9 and Figure 3). Moreover, this cluster contains essential genes needed for learning and memory formation, including the immediate early genes *EGR1*, *ARC*, and *FOS* and several signaling subunits of *PP1*. In addition, this cluster comprises many genes known to play a role in the initiation of critical periods of neuroplasticity, like *BDNF*, *NARP* (*NPTX2*), *GAD1*, and *GAD2*, *CLOCK*, and subunits of the GABA_*A*_ receptor. This illustrates that, between 1 and 20 dph, there is an increase in synaptic signaling and learning potential. Cluster LLL, in contrast, contains 2,913 genes that exhibit a high expression at 1 dph and then decrease to remain constant until adulthood. Functional enrichment analysis showed enrichment of genes involved in cell cycle regulation, nuclear transport, RNA processing, metabolic processes, regulation of gene expression, and chromatin remodeling (Supplementary Table S9 and Figure 3). The high expression of metabolic genes before 20 dph can be explained by the extensive growth and maturation that the brain undergoes between 1 and 20 dph. The decrease in the expression of cell cycle genes most likely reflects a decrease in neuron proliferation, as they transition into a postmitotic state.

Clusters EHH and ELL show transcriptional changes at 65 dph and in adulthood. EHH is enriched for the processes phagocytosis, cell–cell fusion, and glial cell differentiation. ELL contains genes involved in neurodevelopmental regulation of brain vasculature and extracellular matrix organization (Supplementary Table S9 and Figure 3). In the brain, extracellular matrix (ECM) molecules fill the extracellular space and are involved in synaptic stabilization, protection of neurons from oxidative stress, limitation of synaptic plasticity, and closure of developmental critical period plasticity windows (Dzyubenko et al., 2016; Song and Dityatev, 2018). In clusters HEE, LEE, EHE, ELE, EEH, and EEL, gene transcription changes transiently at either 20 or 65 dph or adulthood. HEE shows functional enrichment related to calcineurin-mediated signaling and regulation of intracellular pH, while LEE contains genes related to mRNA processing, RNA splicing, and translation termination. EHE and ELE are involved in glycoprotein metabolic process and protein localization to membrane and in circadian behavior, respectively. EEH is enriched in TOR signaling and O-glycan processing, and EEL in the regulation of protein dephosphorylation and actin filament organization (Supplementary Table S9 and Figure 3).

When studying TFBS enrichment, mainly depletion was observed for the larger gene clusters (HHH, LLL), though always with a limited effect size despite often clear significance (| log FC| < 1; Supplementary Table S10). For some of the smaller clusters, clearly enriched TFBS (| log FC| > 1) could be found; however, the small size of these clusters impeded solid statistical assessment of this enrichment (e.g., TFBS for FOS-like and JUN-like proteins in ELE cluster or E2F-family proteins in EHE cluster, Supplementary Table S10). In the EEL gene cluster, binding motifs for many FOX family transcription factors, including vocal learning-associated genes FOXP1 and FOXP2 (Takahashi et al., 2009; Chen et al., 2013), were about twofold depleted (Supplementary Table S10).

### Age- and Sex-Specific Changes in Telencephalon DNA Methylation During Neurodevelopment

Next to transcription factors, DNA methylation provides another mechanism to regulate gene expression during neurodevelopment. We therefore used (ox)RRBS to study DNA methylation dynamics in the telencephalon during brain development. Methylation changes were detected at 90,113 CpG and 17,805 CpH cytosines, resulting in 24,785 CpG (i.e., 24,551 for the contrast of age and 193, 11, 30, and 0 for the contrast of sex at 1, 20, and 65 dph and adult age, respectively, Supplementary Table S11) and 1,894 CpH (of which 1,835 for the contrast of age and 3, 8, 23, and 25 for the contrast of sex at 1, 20, and 65 dph and adult age, respectively, Supplementary Table S12) differentially methylated cytosines, respectively. For CpG cytosine methylation, this led to 1,861 genes (out of 3,380 genes after filtering) with at least one significantly differentially methylated CpG (of which 1,855 for the contrast of age and 112, 1, 4, and 0 for the contrast of sex at 1, 20, and 65d dph and adult age, respectively). For CpH cytosine methylation, 361 genes were found to be significant (of which 349 for the contrast of age and 1, 2, 11, and 7 for the contrast of sex at 1, 20, and 65 dph and adult age, respectively, out of 1,629 genes after filtering). The general reliability of our results was supported by the observation that significant genes are featured by on average ± 12 significant CpGs and ± 5 significant CpHs covered per gene. These data clearly showed on average lower methylation degrees and, therefore, less differential methylation, in a CpH than in a CpG context, suggesting the latter to be more important for neurodevelopment.

**FIGURE 4:**
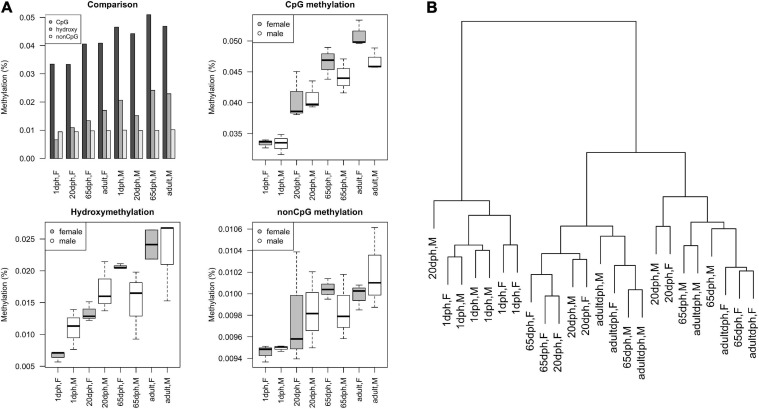
Overview of reduced representation bisulfite sequencing (RRBS) results. **(A)** Methylation levels as a function of age and sex, as found using RRBS, expressed as *M*-values. Comparison of relative CpG methylation, CpG hydroxymethylation, and CpH methylation levels at different ages. Overview of absolute levels of average CpG methylation, hydroxymethylation, and non-CpG methylation levels at different ages in male/female ZF (boxplots). **(B)** Cluster dendrogram displaying hierarchical relationships among RRBS samples. The number of branches separating the samples indicates dissimilarity.

Similar to what has been observed during human brain development (Vogel Ciernia and LaSalle, 2016), postnatal CpG and CpH methylation levels significantly increased for both sets (CpG: *P* = 6.3E-9, CpH: *P* = 0.006) over development (Figure 4A). Remarkably, no significant differences in total methylation between male and female birds could be found (both *P* > 0.13). Along the same line, global CpG hydroxymethylation increased over development (all *P* = 9.4E-5), yet again without significant sex differences (*P* = 0.82, Figure 4A). Likewise, cluster analysis exhibited no clear DNA methylation clustering per sex (Figure 4B). Taking into account that on average observed intensities of CpH were lower than CpG methylation levels, CpG methylation changes seem to be the dominant regulator in neurodevelopment. Moreover, the number of age-specific DNA methylation differences clearly exceeded the number of sex-specific DNA methylation differences.

### CpG and CpH Methylation Regulate Age-Dependent Neurodevelopmental Gene Expression

Next, we focused on those genes for which differential methylation is associated with differential expression. Strikingly, for genes featuring differential expression between sexes, only a single CpG (at 1 dph) and no CpH featured differential methylation; therefore, only age-dependent changes in gene expression will be considered. First, we confirmed the earlier observation that promoter DNA methylation is also involved in gene expression regulation in zebra finch (Steyaert et al., 2016). In general, expression (log-cpm) of age-associated genes was negatively associated with DNA methylation (*M*-values) for both promoter (β = –0.041, *P* < 2E-16) and genic regions (β = –0.046, *P* < 1E-14). Also for CpH methylation, associations were significant for promoter (β = –0.026, *P* = 0.002) as well as genic regions (β = –0.039, *P* < 1E-6).

Subsequently, we considered genes for which both expression and methylation data are available. At the individual cytosine level, limma analysis showed that 24.6% (6,692 out of 27,197) of CpGs and 12.0% (519 out of 4,325) of CpHs analyzed in genes differentially expressed with age displayed a significantly changed methylation status over telencephalon development (Supplementary Tables S13, S14). Vice versa, 34.1 and 52.1% of genes differentially expressed with age show at least one differentially methylated CpG in the promoter and genic regions, respectively. However, for age, there was no significant enrichment in differential promoter methylation for the differentially expressed genes compared with the non-differentially expressed genes (*P* = 0.80). This result implies that DNA methylation cannot be considered a global driver of age-dependent gene expression changes.

**FIGURE 5:**
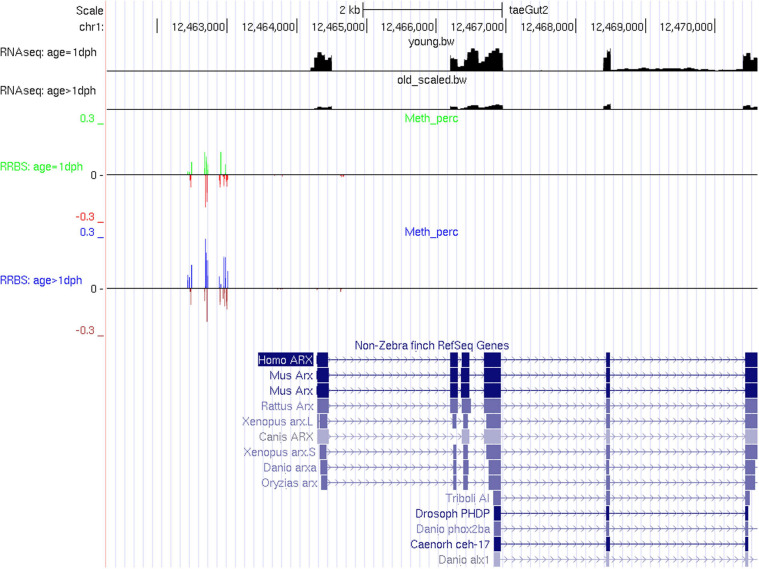
RNA-Seq and RRBS tracks for the gene ARX. In the RRBS tracks, the average methylation rate is provided as a positive number for significantly methylated CpGs and as a negative number for insignificantly methylated CpGs.

However, expression of individual key neurodevelopmental genes may be tightly controlled by DNA methylation, as illustrated here for *ARX* (Figure 5). *ARX* is a homeobox transcription factor gene essential for normal telencephalon development (Sherr, 2003), known to be regulated by DNA methylation in humans (Dhawan et al., 2011) and previously found to be upregulated upon chemical demethylation in zebra finch (Steyaert et al., 2016). Out of 21 significantly differentially methylated CpGs, 19 show hypermethylation with age, coinciding with a highly significant reduction in expression (4.11-fold reduction, adj. *P* = 2.9E-257). For further qualitative results, UCSC tracks containing RNA-Seq data and methylation percentages for CpGs within promoter or genic regions of differentially expressed genes can be found in Supplementary Files S1–S4. Functional enrichment analysis of differentially expressed genes containing one or more differentially methylated CpGs or CpHs revealed enrichment for biological processes related to pattern specification, regionalization, embryonic organ development, sensory organ development, developmental growth, and axonogenesis (Supplementary Table S15 and Supplementary Figure S3).

Strikingly, enrichment analysis of GO molecular functions of age-specific differentially expressed genes containing differentially methylated CpGs or CpHs revealed a very strong enrichment for regulation of DNA-binding transcription factor activity. Indeed, many of these genes are transcription factors, which could in turn control downstream target gene expression (Supplementary Table S15). For no less than 67.4% of the differentially expressed TFs, also differentially methylated CpGs were found, compared with only 36.3% for non-TF (*P* = 1.2E-9). Important examples include master regulators of critical period synaptic plasticity, i.e., *OTX2*, *AR*, and *FOS* (Supplementary Tables S13, S14) (Gandolfi et al., 2017; Apulei et al., 2019; Tozzi et al., 2019).

Next, we also evaluated the genomic neighborhood of cytosines differentially methylated with age for TFBS enrichment. These analyses demonstrated for example a clearly significant enrichment for *ESRRA* binding motifs (∼2-fold, *P* = 4.8E-5) (Supplementary Data Sheet 1), suggesting that the transactivation activity of *ESRRA* may depend on the DNA methylation status of its binding motif. Moreover, binding motifs for *EGR1*, *EGR2*, *EGR3*, and *EGR4*, all members of the early growth response (EGR) family of immediate early gene transcription factors, were depleted. This gene family is rapidly and transiently activated in response to a wide range of stimuli and can translate environmental influence into long-term changes in the brain, contributing to neuronal plasticity (Pfaffenseller et al., 2018). Interestingly, *EGR1* was found to recruit Tet1 to shape the brain methylome during development and upon neuronal activity (Sun et al., 2019). In total, we found at least a twofold depletion or enrichment of 67 TFBS, suggesting a thorough interplay between both DNA methylation and TF transactivation (Supplementary Data Sheet 1; Banerjee et al., 2019).

In summary, whereas promoter methylation is generally associated with gene silencing, it is not sufficient to explain the massive differential gene expression observed with age in the zebra finch telencephalon. However, differential methylation was preferentially observed in TFs differentially expressed with age, which in turn target various neurodevelopmental genes differentially expressed across ages. Reciprocally, regions around CpGs differentially methylated across ages show clear enrichment for several TFBS. In this respect, our results reveal important reciprocal control mechanisms of DNA methylation and TF activity during neurodevelopment.

### Immunofluorescence Reveals Elevated 5mC Levels in the Song Control System

**FIGURE 6:**
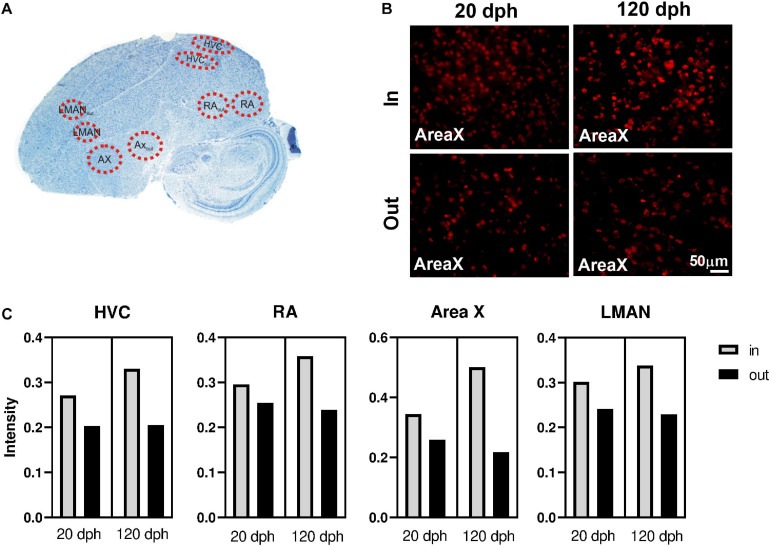
Immunofluorescence of 5-methylcytosine (5mC) in zebra finch telencephalon at 20 and 120 dph. **(A)** Overview of the analyzed regions on a Nissl-stained sagittal section of a 120-dph zebra finch brain. **(B)** 40× photomicrographs of Area X and its surrounding region (Area X_out_) in brain sections of a 20- and a 120-dph old zebra finch, as an example of the 5mC-specific antibody staining used for intensity measurements. **(C)** Average 5mC intensity per cell nucleus in HVC, RA, Area X, and LMAN at 20 and 120 dph. Raw means were calculated as the mean intensity of the cells within the region considered, averaged over the four birds for that time point.

Since we used whole telencephalon samples, our results lack detailed spatial information. We hypothesized that DNA methylation could be particularly relevant in learning related brain regions, like the song control nuclei, compared with surrounding tissue. Therefore, we used 5mC-specific immunofluorescence on two relevant time points (20 and 120 dph, four males each), to compare 5mC levels inside the four major song control nuclei—Area X, HVC, RA, and LMAN—to equally large areas just outside of these nuclei (Area X_out_, HVC_out_, RA_out_, and LMAN_out_, together SCS_out_). A schematic representation of the selected areas is shown on a Nissl-stained zebra finch brain section (Figure 6A), and representative examples for Area X immunofluorescence images are depicted in Figure 6B.

Per individual brain region (Area X, HVC, RA, and LMAN), we used a linear mixed model to evaluate whether the 5mC signal intensity per cell (as proxy for the degree of DNA methylation) varied as a function of time point (20 and 120 dph) and position (“inside/outside” of the song control nucleus), as well as their interaction, adjusted for brain slice as random factor (Table 1). For each brain region, 5mC signal intensity was significantly higher inside the song control nucleus than in the corresponding region outside of the nucleus (all *P* < 0.0005). Whereas the main effect of time point was never significant (all *P* > 0.6), for each brain region, there was a significant interaction between “inside/outside” and time point (all *P* < 0.002), indicating a further increase with age of methylation in the song control nuclei, but not in cells outside of the song nuclei (Figure 6C). Nevertheless, additional work is required to confirm the relevance of 5mC in song learning by measuring gene-specific DNA methylation differences specifically in the song control nuclei.

**TABLE 1:** Evaluation of 5mC immunofluorescence signal intensity per cell as a function of time point (20 and 120 dph) and position (“inside/outside” of the song control nucleus), as well as their interaction, adjusted for brain slice as random factor, using a linear mixed model per brain region (HVC, RA, Area X, LMAN).

	HVC		RA		AX		LMAN	
	Estimate (SEM)	*P*-value	Estimate (SEM)	*P*-value	Estimate (SEM)	*P*-value	Estimate (SEM)	*P*-value
Intercept (day = 20; outside the song control nucleus)	0.202 (0.026)	1.74E-04	0.254 (0.026)	3.16E-05	0.258 (0.058)	4.00E-03	0.241 (0.027)	7.56E-05
Effect day 120	0.002 (0.037)	9.55E-01	−0.015 (0.036)	6.83E-01	−0.040 (0.081)	6.36E-01	−0.015 (0.036)	7.59E-01
Effect inside the song control nucleus	0.069 (0.010)	9.88E-11	0.041 (0.012)	3.91E-04	0.085 (0.011)	6.85E-14	0.060 (0.011)	8.16E-08
Interaction: effect inside the control nucleus, day 120	0.056 (0.015)	1.33E-04	0.078 (0.016)	2.69E-06	0.198 (0.015)	2.56E-29	0.049 (0.016)	1.88E-03

### Sexually Dimorphic Gene Expression

As already mentioned in previous paragraphs, about half of all genes featuring a significant sex difference between males (ZZ) and females (ZW) are located on the Z chromosome. Genes differentially expressed between sexes at every time point are almost exclusively found on the Z chromosome (97.0% out of 167 genes), whereas genes that are specifically differentially expressed at a single time point tend to be less frequently linked to the Z chromosome (19.2, 5.1, 12.8, and 1.1%, out of 224, 136, 149, and 94 genes, for resp. 1, 20, and 65 dph and adults). This implies that sex-dependent differences in gene expression arising after 1 dph are mainly (90.5%) autosomal in origin. As a net result, from young to old, respectively, 69.3, 71.6, 72.7, and 59.9% of the genes differentially expressed between sexes were located on the Z chromosome. This fraction is significantly lower for adults compared with other time points (all adj. *P* < 0.04), whereas other differences were non-significant (all adj. *P* = 1).

As expected, Z-linked differentially expressed genes particularly show overexpression in male birds (average over four time points: 99.0%, with no differences in directionality between time points; *P* = 0.82). For autosomal genes on the other hand, the three youngest groups exhibit depletion of overexpressed genes for male birds (average over three time points: 31.3%, no differences between these time points; all adj. *P* > 0.62). Strikingly, for adult birds, there was a significant enrichment of autosomal gene overexpression in male birds (67.6%, all adj. *P* < 1.7E-7), yet less pronounced compared with Z-linked genes.

### Incomplete Dosage Compensation in Zebra Finch Brain

**FIGURE 7:**
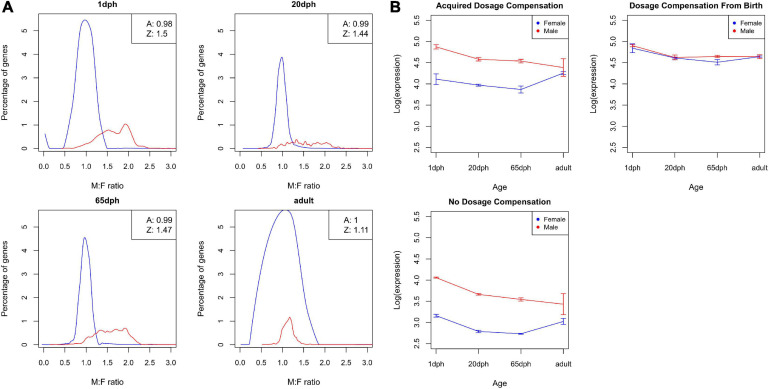
Dosage compensation. **(A)** Distribution of male-to-female (M:F) ratios of gene expression for zebra finch telencephalon samples at four different ages: post-hatch days 1, 20, and 65 and adult. The distribution for autosomal genes is shown as a black line, the distribution for Z genes as a red line. The boxes in the upper right corner show the average M:F ratios for autosomal and Z chromosome genes. **(B)** Average log-transformed cpm values over different ages for both male birds (red) and female birds (blue) for (i) genes that acquired dosage compensation, (ii) genes that are dosage compensated from birth, and (iii) non-dosage compensated genes.

Sex-specific expression differences in Z chromosome-linked genes reflect incomplete dosage compensation in birds (Ellegren et al., 2007; Itoh et al., 2007, 2010). In order to evaluate whether the degree of dosage compensation varies throughout development, we evaluated M:F expression ratios. As shown in Figure 7A, the distribution of M:F ratios for autosomal genes is centered at 1, i.e., no sex bias, whereas an overall male bias in expression was observed for Z genes (M:F ratios of 1.50, 1.44, 1.47, and 1.11 for 1, 20, and 65 dph and adult, respectively, a ratio of 1 or 2 implies complete compensation vs. no compensation). These results clearly confirm incomplete dosage compensation in zebra finch, accounting for a large part of the observed sex differences. Moreover, the degree of dosage compensation largely varied between genes, with 95% confidence interval of Z gene M:F ratios ranging from 0.90 to 2.28. Strikingly, with an average ratio of 1.11, dosage compensation of Z genes is far higher in adult samples compared with the other time points and even approximates complete dosage compensation (Figures 7A,B).

Subsequently, we grouped Z genes into three categories: genes that are dosage compensated from birth (*n* = 78), genes that become compensated throughout life (*n* = 428), and genes that never become dosage compensated (*n* = 132) (see section “*Materials and Methods,”* Supplementary Data Sheet 2). Unexpectedly, dosage compensated genes exhibited on average higher expression levels than non-dosage compensated ones (both adj. *P* < 6.9E-5, Figure 7B). Even more strikingly, for genes that acquire dosage compensation during life, compensation is partly driven by a decrease in expression in the homogametic male (cf. mammals), but also partly due to an increase in expression with age in females (Figures 7A,B).

Functional enrichment analysis showed enrichment for neurotransmitter (serotonin) and metabolic processes (oxidative phosphorylation, lipidation) in genes that are never dosage compensated (Supplementary Figure S4 and Supplementary Data Sheet 2). Genes that acquire dosage compensation throughout life exhibited enrichment for ciliary neurotrophic factor (CNTF)-mediated signaling (Supplementary Figure S4 and Supplementary Data Sheet 2). Interestingly, CNTF signaling in ciliated sensory neurons plays key developmental roles in postnatal brain maturation of sex-specific behavior (Morris et al., 2004; Koemeter-Cox et al., 2014; Jia et al., 2019). Additionally, TFBS enrichment analysis was performed on dosage compensated genes from birth onwards and genes that become dosage compensated later in life (Supplementary Data Sheet 3), compared with non-dosage compensated genes. Genes that were dosage compensated from birth onwards only showed two depleted groups of (neuro)developmental TFBS (i.e., Ets-related factors *ERF* and *ERG* and RXR-related receptors *RXRB* and *RXRG*). Genes that acquire dosage compensation later in life showed a twofold motif enrichment (*P* < 0.006) for *TBX19*, known to regulate developmental adenocorticotropic hormone functions (Abali et al., 2019).

## Discussion

Neural circuits are shaped by experience during periods of heightened brain plasticity in early postnatal life, called critical periods. Song learning in zebra finches is one of the most striking examples of a critical period for complex learned behavior (Kelly et al., 2018); moreover, this behavior as well as the underlying brain circuit is sexually dimorphic. Through genome-wide molecular characterization of the neurodevelopmental epigenomic and transcriptomic dynamics of the zebra finch telencephalon from 1 dph to adulthood, we provide novel insights into possible molecular determinants of age- and sex-dependent gene expression dynamics in the developing zebra finch brain. Moreover, generated matched expression and methylation data (GEO ID: GSE147974), as well as relevant summarized data, are made publicly available as community resource for further study (e.g., UCSC-compatible Supplementary bedGraph and BigWig Files S1–S4).

We identified major age- and sex-specific transcriptional changes in neurodevelopmental gene expression. Epigenetic investigation of global CpG methylation, non-CpG (CpH) methylation, and CpG hydroxymethylation levels over brain development using (ox)RRBS revealed that the levels of each of these modifications were low, but increased steadily during telencephalon development. A similar initial low degree combined with a global increase in DNA methylation has been observed during human brain development (Lister et al., 2013; Dillman and Cookson, 2014; Vogel Ciernia and LaSalle, 2016) and may reflect the role of methylation in experience-dependent shaping of the neuronal transcriptome. Along the same line, immunofluorescent staining of methylcytosine in the brain revealed an age-dependent increase in signal intensity specifically in the song control system during the period of song learning. Furthermore, this suggests that DNA methylation is particularly relevant for the SCS and possibly song learning.

Since the investigated time points span the entire period of zebra finch brain maturation, innumerable functional and structural changes were expected to occur. Indeed, gene classification according to similar developmental expression patterns identified coordinated, large-scale cellular changes during brain development, some of which show strong enrichment for specific TF binding motifs. Only a limited fraction of the massive differential expression in the developing zebra finch telencephalon could be explained by differential CpG and/or CpH DNA methylation. However, a strong association between DNA methylation and age-dependent gene expression was found for various transcription factors involved in neurodevelopment. Additionally, we found significant enrichment of several TF binding motifs around differentially methylated cytosines in differentially expressed genes, suggesting that DNA methylation controls the binding of transcription factors to their binding sites (Kribelbauer et al., 2017, 2019; Yin et al., 2017; Heberle and Bardet, 2019). Together, these results indicate that DNA methylation regulates neurodevelopmental gene expression dynamics through steering transcription factor activity (i.e., *ESRRA*, *FOS*, *OTX2*, *AR*, *EGR* family) rather than by regulating all individual genes.

In accordance with previous studies showing that dosage compensation in birds is much more limited than in mammals (Ellegren et al., 2007; Itoh et al., 2007, 2010), we found that incomplete dosage compensation of Z chromosome genes was largely responsible for sexually dimorphic gene expression. Only a smaller number of sex differences arising after 1 dph are largely autosomal in origin. Z-linked genes were grouped into three categories: genes that are dosage compensated from birth, genes that become compensated throughout life, and genes that never become dosage compensated. The majority of sex-biased genes are not dosage compensated at 1 dph, but become dosage compensated throughout life, meaning that they can contribute to sex-specific patterns in development, even though they are balanced in adulthood. Indeed, dosage compensation of Z chromosome genes was found to be far higher in adult samples, compared with all the other time points, and even approximated complete dosage compensation. Interestingly, for genes that acquire dosage compensation during life, compensation is partly driven by a decrease in expression in the homogametic male, but also partly due to an increase in expression with age in females. Finally, in contrast to placental mammals where DNA methylation is one of the inhibitory mechanisms regulating dosage compensation by X chromosome inactivation (Payer and Lee, 2008), we found no evidence for a role of DNA methylation in dosage compensation in zebra finches.

Previously, it has been shown that brain feminization in mammals requires active suppression of masculinization *via* DNA methylation (Nugent et al., 2015). In contrast, our results show no significant sex difference in global methylation levels. Also, at the single gene level, we only identified major neurodevelopmental age- but not sex-specific CpG/CpH gene methylation changes in differentially expressed genes. These results indicate that DNA methylation is not involved in regulating sexually differentiated gene expression. However, we cannot exclude a possible role for CpG/CpH DNA methylation in regulating (sexually differentiated) alternative RNA splicing and isoform use (Blekhman et al., 2010; Angelova et al., 2018; Shayevitch et al., 2018; Linder and Jaffrey, 2019; Price et al., 2019).

One limitation of our study concerns the heterogeneity of our telencephalon samples, which contains diverse brain regions and many different cell types. Our results represent an average over all cell types and may mask gene expression patterns and epigenetic changes occurring in cell populations with low abundance, or in specific brain nuclei (Lovell et al., 2018). In the future, this could be overcome using currently emerging single-cell sequencing and epigenomics technologies, which would allow to study gene expression and methylome in specific brain cell types (Karemaker and Vermeulen, 2018). A second limitation is that RRBS does not cover the whole genome, and relevant methylation changes may be missed. In addition, RRBS does not allow mapping of newly discovered DNA and RNA N6-methyladenine modifications involved in neurodevelopment or sex determination (Kigar et al., 2017; Yao et al., 2017, 2018; Livneh et al., 2020). This may now be overcome by nanopore and long read single molecule real-time (SMRT) sequencing approaches (Chen et al., 2019; Gouil and Keniry, 2019; Liu et al., 2019; Pacini et al., 2019). Third, due to a lack of comprehensive avian TF binding site annotation, we used human annotations for TF enrichment analysis. Even though TF binding sites are well conserved (Nitta et al., 2015), we could have missed some species-specific TF enrichment. Finally, the used version of the Zebra Finch genome (taeGut3.2.4) was based on male sequences solely. Apart from the inability to map transcripts to the W chromosome, this could cause some additional mapping problems due to W/Z pseudoautosomal regions.

In conclusion, our results indicate that DNA methylation regulates neurodevelopmental gene expression dynamics through steering relevant transcription factor activity, but does not explain sex-specific gene expression patterns in zebra finch telencephalon, nor does it seem to be involved in the regulation of dosage compensation in zebra finches.

## Data Availability Statement

The datasets presented in this study can be found in online repositories. The names of the repository/repositories and accession number(s) can be found in the article/Supplementary Material.

## Ethics Statement

The animal study was reviewed and approved by the Ethical Committee for Animal Experimentation at the University of Liège (protocol 1396) and University of Antwerp (ECD 2016-70).

## Author Contributions

JD wrote the manuscript text, with the help from LC, under the supervision of WVB and TD. JD, LC, TD, WVB, JB, WVC, AV, and CC contributed to the conception and design of the experiments. AV, CF-V, WVC, WVB, and TD supervised the research. JD, LC, WVC, and TD contributed to the interpretation of the results. JD, ED, SD, and GM performed the laboratory experiments. LC and SS performed (bio)informatic and statistical analyses. JD and WVB performed functional enrichment analysis. CF-V assisted with the functional analysis of the data. LC and JD made the figures. JG was responsible for data preprocessing and data management. SH collected the brain samples under the supervision of CC and JB. AV and WVC acquired funding. All authors reviewed the manuscript.

## Conflict of Interest

The authors declare that the research was conducted in the absence of any commercial or financial relationships that could be construed as a potential conflict of interest.

## Footnotes

1 http://metascape.org/

## Supplementary Material

Supplementary Figure 1 Cluster dendrogram displaying hierarchical relationships among RNAseq samples.

Supplementary Figure 2 Difference between oxidative RRBS and total RRBS signal. The difference between oxidative RRBS and total RRBS signal, i.e., hydroxymethylation signal, showing a normal distribution around zero, indicating difficulties for separating signal from noise.

Supplementary Figure 3 Top Biological processes in genes differentially expressed over development and regulated by CpG/CpH methylation. Based on GePS functional enrichment analysis. GO-Terms are sorted by *P*-value for CpG positions. For similar GO terms, only the top-ranked GO term was shown. The dot size reflects the number of genes enriched in the GO term and its color the corresponding statistical significance (*P*-value). Its absence indicates the annotation is not significantly enriched for the given condition. Rich Factor is the ratio of differentially expressed gene numbers annotated in this GO term to all gene numbers annotated in this GO term. Complete enrichment analysis results are included in Supplementary Table S15.

Supplementary Figure 4 Top GO Biological processes for Z-linked genes depending on their dosage compensation. Z-linked genes were grouped as follows: never dosage compensated (no), dosage compensated from birth (birth), or acquired dosage compensation later in life (acquired). Figure based on GePS functional enrichment analysis. GO-Terms are sorted by *P*-value in GePS functional enrichment analysis. The dot size reflects the number of genes enriched in the GO term and its color the corresponding statistical significance (*P*-value). Rich Factor is the ratio of differentially expressed gene numbers annotated in this GO term to all gene numbers annotated in this GO term. Complete enrichment analysis results are included in Supplementary Data Sheet 2.

Supplementary Table 1 RRBS-sequencing protocol.

Supplementary Table 2 Summary statistics RRBS.

Supplementary Table 3 Summary statistics RNA-seq.

Supplementary Table 4 Full output RNA-seq analysis.

Supplementary Table 5 Functional enrichment analysis for differentially expressed genes for effect of sex, using genomatix and metascape (available online via https://doi.org/10.5281/zenodo.4278907 or http://biobix.be/plastoscine/).

Supplementary Table 6 Summary of age and sex specific changes in expression of DNA-RNA writer-reader-eraser proteins and histone variants.

Supplementary Table 7 TFBS analysis for differentially expressed genes for the effect of age or sex.

Supplementary Table 8 RNA-seq analysis results for 12 gene expression pattern clusters.

Supplementary Table 9 Genomatix and metascape functional enrichment analysis for different gene expression pattern clusters.

Supplementary Table 10 TFBS enrichment analysis for different gene expression pattern clusters.

Supplementary Table 11 Full output for the RRBS analysis for CpG methylation (available online via https://doi.org/10.5281/zenodo.4278907 or http://biobix.be/plastoscine/).

Supplementary Table 12 Full output for the RRBS analysis for non-CpG (CpH) methylation.

Supplementary Table 13 RRBS output for CpG methylation in differentially expressed genes.

Supplementary Table 14 RRBS output for non-CpG (CpH) methylation in differentially expressed genes.

Supplementary Table 15 Functional enrichment analysis for genes differentially expressed and methylated during neurodevelopment.

Supplementary Data Sheet 1 TFBS analysis for flanking regions of age dependent differentially methylated CpG dinucleotides.

Supplementary Data Sheet 2 Gene lists and functional enrichment of Z-linked genes according to timing of dosage compensation.

Supplementary Data Sheet 3 TFBS analysis of dosage compensated genes (from birth or acquired), as compared to non-dosage compensated genes.

Supplementary File 1 Bedgraph track for average methylation percentage of CpG dinucleotides for birds at the age of 1 dph (available online via https://doi.org/10.5281/zenodo.4278907 or http://biobix.be/plastoscine/). These bedgraph tracks are compatible with the UCSC genome browser. The average methylation rate is provided as a positive number for significantly methylated CpGs and as a negative number for insignificantly methylated CpGs.

Supplementary File 2 Bedgraph track for average methylation percentage of CpG dinucleotides for birds older than 1 dph (available online via https://doi.org/10.5281/zenodo.4278907 or http://biobix.be/plastoscine/). These bedgraph tracks are compatible with the UCSC genome browser. The average methylation rate is provided as a positive number for significantly methylated CpGs and as a negative number for insignificantly methylated CpGs.

Supplementary File 3 BigWig track for RNAseq data for birds at the age of 1 dph (available online via https://doi.org/10.5281/zenodo.4278907 or http://biobix.be/plastoscine/). These bigWig tracks are compatible with the UCSC genome browser. The coverage is provided in binned regions of 50 basepairs (default). Tracks were scaled so both RNAseq tracks are comparable considering different sample sizes.

Supplementary File 4 BigWig track for RNAseq data for birds older than 1 dph (available online via https://doi.org/10.5281/zenodo.4278907 or http://biobix.be/plastoscine/). These bigWig tracks are compatible with the UCSC genome browser. The coverage is provided in binned regions of 50 basepairs (default). Tracks were scaled so both RNAseq tracks are comparable considering different sample sizes.

## Abbreviations

5mC: 5-methylcytosine
cpm: counts per million
dph: days post hatch
EGR: early growth response
FC: fold change
FDR: false discovery rate
GePS: Genomatix Pathways System
GO: Gene Ontology
IF: immunofluorescence
LMAN: lateral magnocellular nucleus of the nidopallium
oxRRBS: oxidative RRBS
PWM: probability weight matrices
RA: robust nucleus of the arcopallium
RRBS: reduced representation bisulfite sequencing
SCS: song control system
TF: transcription factor
TFBS: transcription factor binding site.
