# Supplementary figures and images for: DNA Methylation Regulates Transcription Factor-Specific Neurodevelopmental but Not Sexually Dimorphic Gene Expression Dynamics in Zebra Finch Telencephalon
Source: Front Cell Dev Biol. 2021 Mar 19;9:583555. doi: 10.3389/fcell.2021.583555 (PMC8017237; doi:10.3389/fcell.2021.583555)

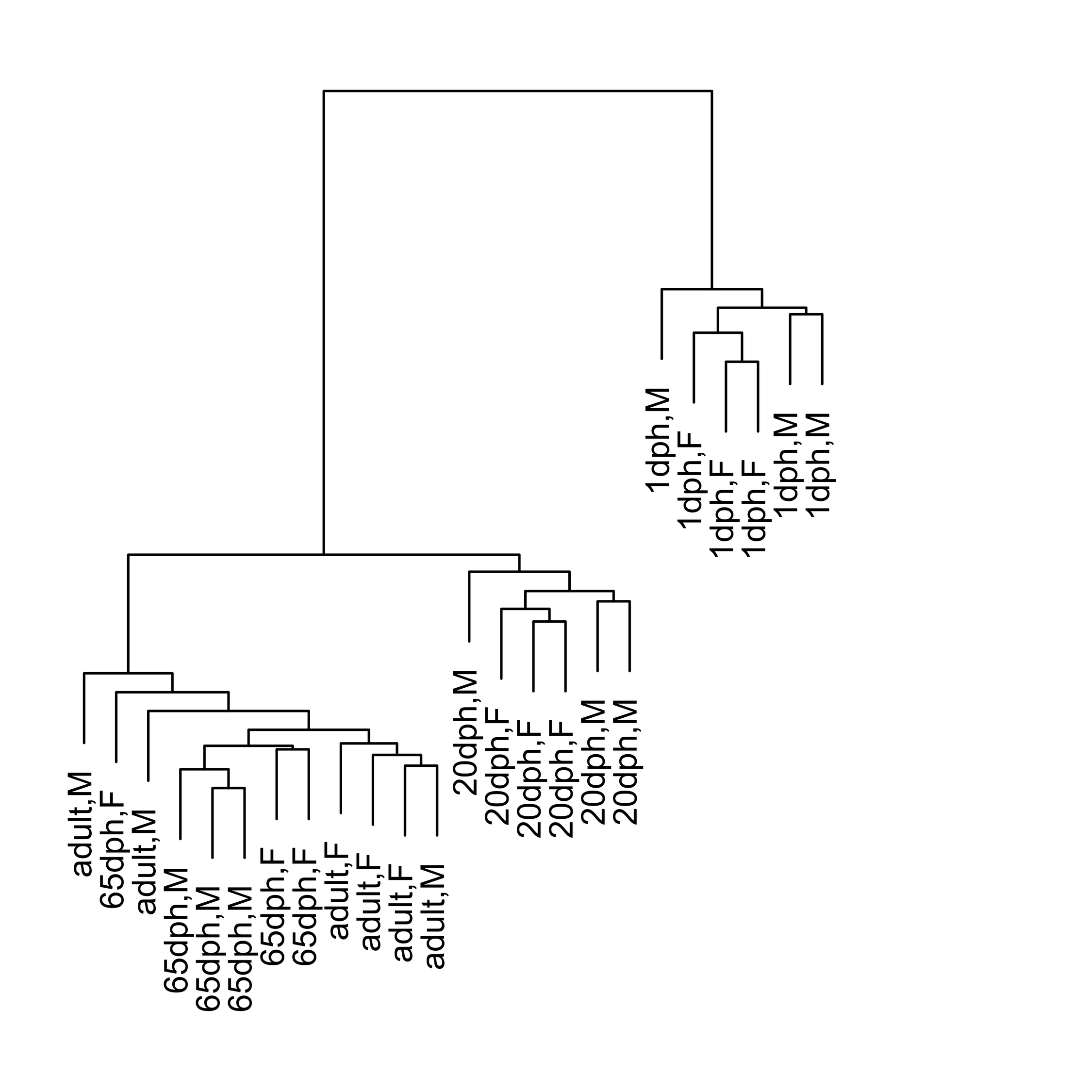

Supplement: Supplementary Figure 1 — Cluster dendrogram displaying hierarchical relationships among RNAseq samples. [file Image_1.TIF]

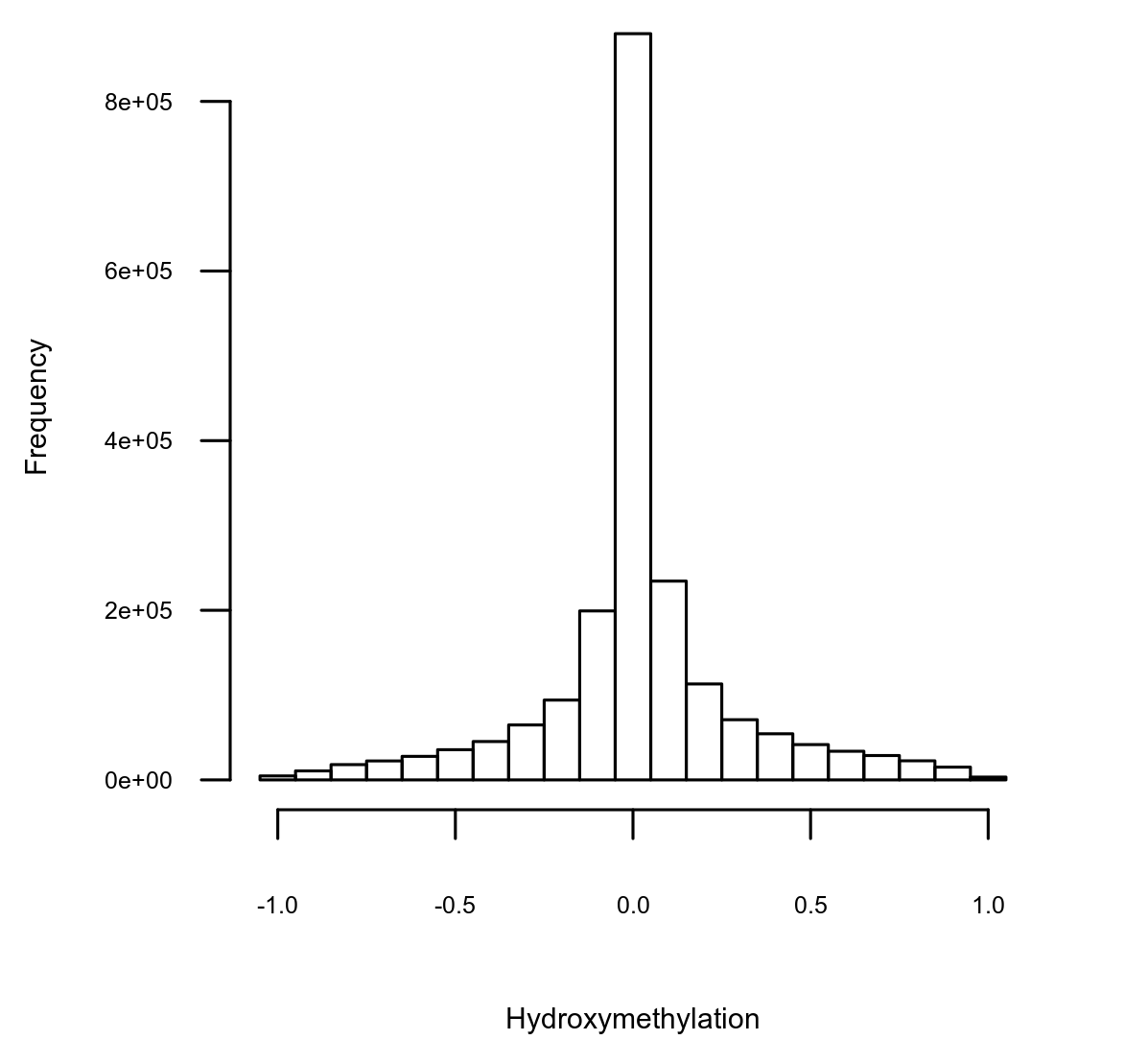

Supplement: Supplementary Figure 2 — Difference between oxidative RRBS and total RRBS signal. The difference between oxidative RRBS and total RRBS signal, i.e., hydroxymethylation signal, showing a normal distribution around zero, indicating difficulties for separating signal from noise. [file Image_2.TIF]

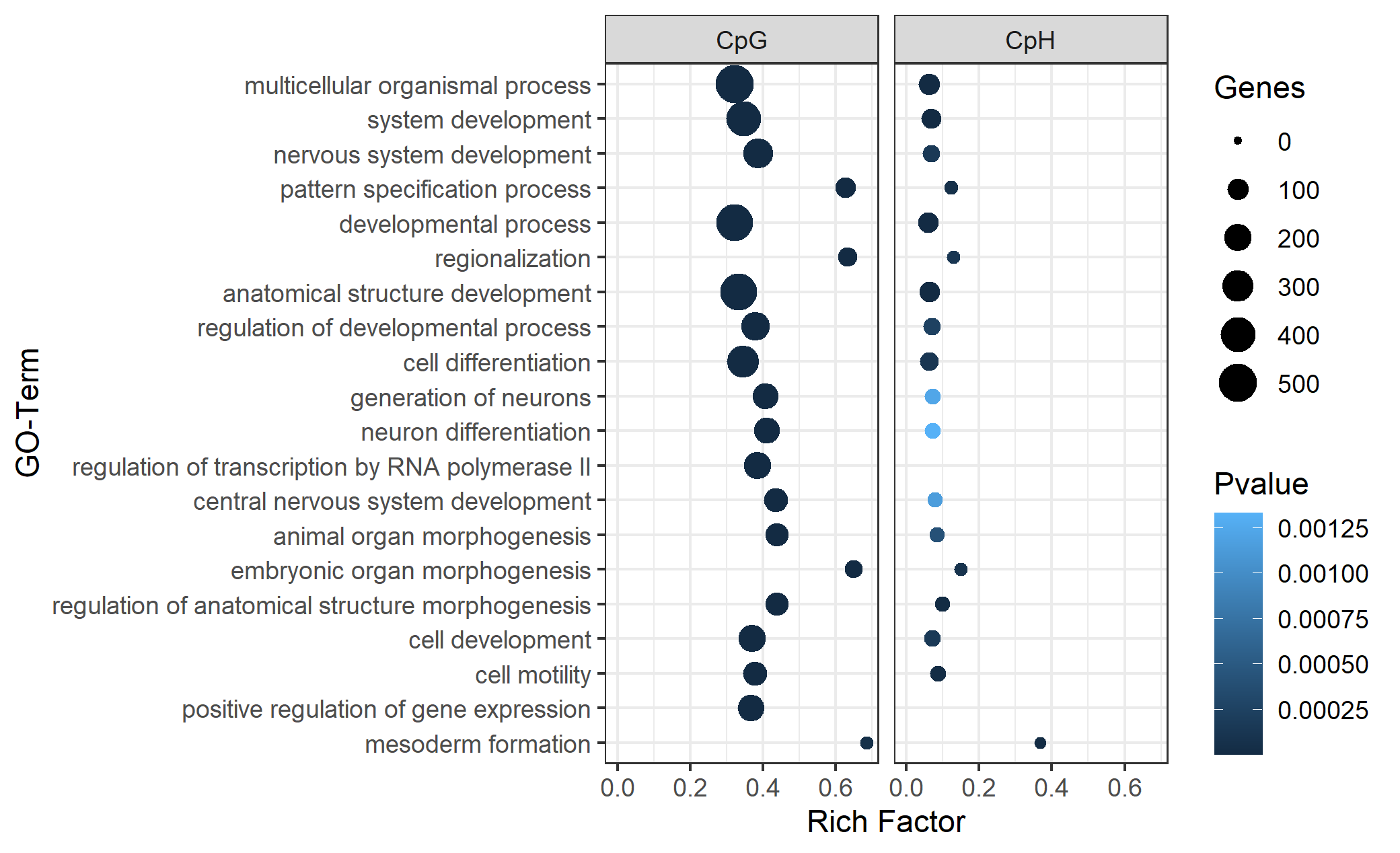

Supplement: Supplementary Figure 3 — Top Biological processes in genes differentially expressed over development and regulated by CpG/CpH methylation. Based on GePS functional enrichment analysis. GO-Terms are sorted by P-value for CpG positions. For similar GO terms, only the top-ranked GO term was shown. The dot size reflects the number of genes enriched in the GO term and its color the corresponding statistical significance (P-value). Its absence indicates the annotation is not significantly enriched for the given condition. Rich Factor is the ratio of differentially expressed gene numbers annotated in this GO term to all gene numbers annotated in this GO term. Complete enrichment analysis results are included in Supplementary Table S15. [file Image_3.TIF]

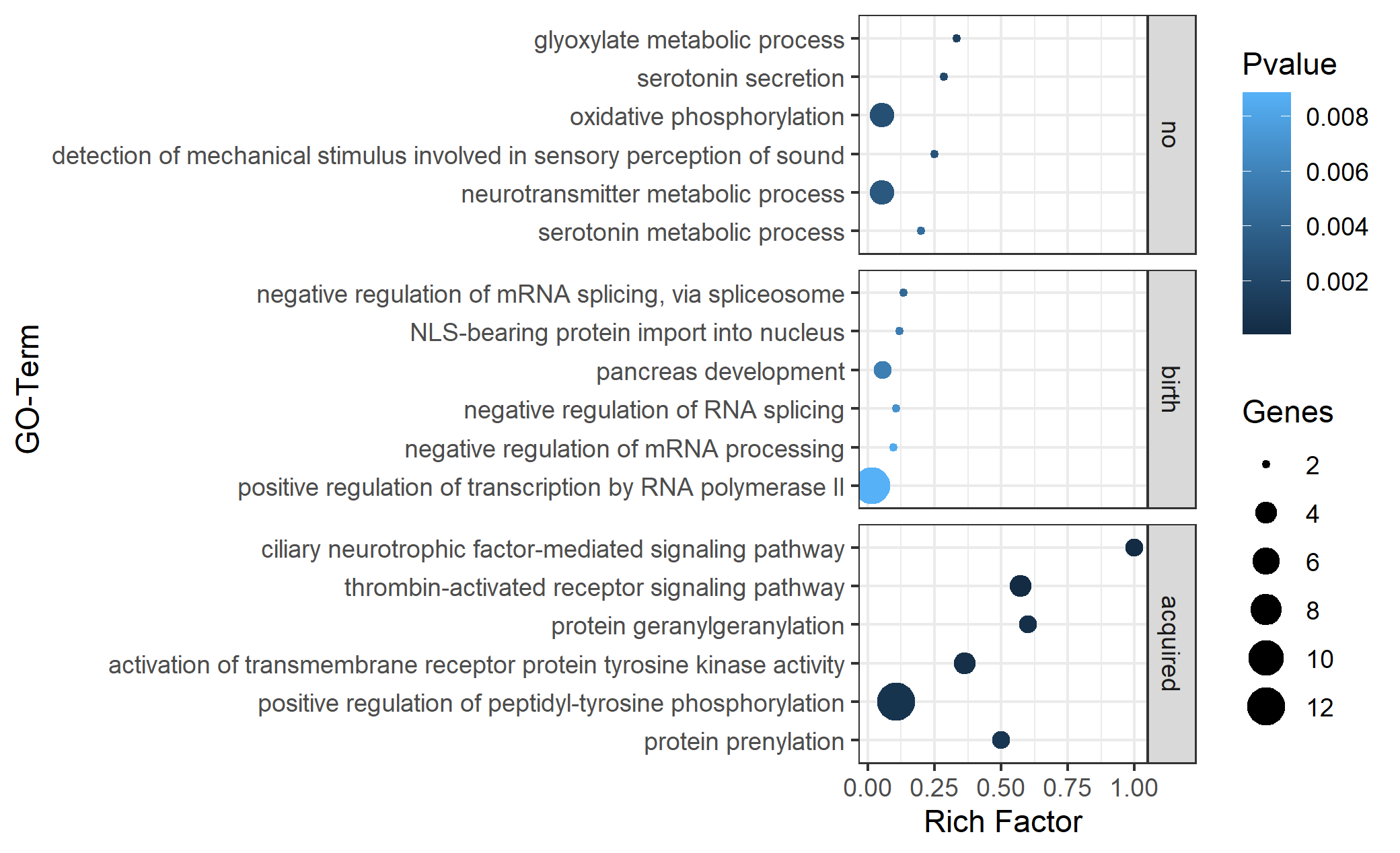

Supplement: Supplementary Figure 4 — Top GO Biological processes for Z-linked genes depending on their dosage compensation. Z-linked genes were grouped as follows: never dosage compensated (no), dosage compensated from birth (birth), or acquired dosage compensation later in life (acquired). Figure based on GePS functional enrichment analysis. GO-Terms are sorted by P-value in GePS functional enrichment analysis. The dot size reflects the number of genes enriched in the GO term and its color the corresponding statistical significance (P-value). Rich Factor is the ratio of differentially expressed gene numbers annotated in this GO term to all gene numbers annotated in this GO term. Complete enrichment analysis results are included in Supplementary Data Sheet 2. [file Image_4.TIF]
